# Obacunone alleviates ferroptosis during lipopolysaccharide-induced acute lung injury by upregulating Nrf2-dependent antioxidant responses

**DOI:** 10.1186/s11658-022-00318-8

**Published:** 2022-03-19

**Authors:** Jin Li, Shi-hua Deng, Jing Li, Li Li, Feng Zhang, Ye Zou, Dong-ming Wu, Ying Xu

**Affiliations:** 1 The First Affiliated Hospital of Chengdu Medical College, Chengdu, Sichuan 610500 People’s Republic of China; 2 School of Clinical Medicine, Chengdu Medical College, Chengdu, Sichuan 610500 People’s Republic of China

**Keywords:** Obacunone, Nrf2, Ferroptosis, Acute lung injury, Lipopolysaccharide

## Abstract

**Background:**

Acute lung injury (ALI) has received considerable attention in the field of intensive care as it is associated with a high mortality rate. Obacunone (OB), widely found in citrus fruits, is a natural bioactive compound with anti-inflammatory and antioxidant activities. However, it is not clear whether OB protects against lipopolysaccharide (LPS)-induced ALI. Therefore, in this study, we aimed to evaluate the protective effects of OB and the potential mechanisms against LPS-induced ALI and BEAS-2B cell injury.

**Methods:**

We established a model of BEAS-2B cell injury and a mouse model of ALI by treating with LPS. Samples of in vitro model were subjected to cell death, Cell Counting Kit-8, and lactate dehydrogenase (LDH) release assays. The total number of cells and neutrophils, protein content, and levels of IL-6, TNF-α, and IL-1β were determined in bronchoalveolar lavage fluid (BALF). Glutathione, reactive oxygen species, and malondialdehyde levels were determined in lung tissue. Additionally, immunohistochemical analysis, immunofluorescence, western blot, quantitative real-time PCR, and enzyme-linked immunosorbent assay were conducted to examine the effects of OB. Furthermore, mice were treated with an Nrf2 inhibitor (ML385) to verify its role in ferroptosis. Data were analyzed using one-way analysis of variance or paired *t*-tests.

**Results:**

Compared with the LPS group, OB effectively alleviated LPS-induced ALI by decreasing lung wet/dry weight ratio, reactive oxygen species and malondialdehyde production, and superoxide dismutase and glutathione consumption in vivo. In addition, OB significantly alleviated lung histopathological injury, reduced inflammatory cytokine secretion and Fe^2+^ and 4-HNE levels, and upregulated GPX4, SLC7A11, and Nrf2 expression. Mechanistically, OB activated Nrf2 by inhibiting Nrf2 ubiquitinated proteasome degradation. ML385 reversed the protective effects of OB against LPS-induced ALI.

**Conclusion:**

Overall, OB alleviates LPS-induced ALI, making it a potential novel protective agent against LPS-induced ALI.

## Background

Acute lung injury (ALI) is a severe respiratory system disease with global prevalence; it is induced by both endogenous and exogenous pathogenic factors. It mainly manifests as uncontrolled oxidative stress, pulmonary edema, and inflammatory cell infiltration [1]. Lipopolysaccharide is considered an effective activator of the innate immune response by activating the TLR4 pathway [2] and can induce the production of inflammatory mediators and reactive oxygen species (ROS) [3, 4]. ALI, which may subsequently lead to the development of acute respiratory dysfunction syndrome (ARDS) in critical conditions, is one of the main causes of death in critical patients. The pathogenesis of ALI has not been clearly elucidated. Currently, its clinical treatment mainly includes protective mechanical ventilation, with no effective drug for its treatment [5, 6]. Therefore, it is important to elucidate the detailed pathogenesis and pathophysiological mechanisms of ALI and develop effective drugs for its treatment.

Recent studies have shown that ferroptosis occurs during ALI. Ferroptosis is an iron-dependent, lipid-peroxidation-driven cell death cascade. It is different from apoptosis, autophagy, and other forms of cell death, and is mainly characterized by the production of large quantities of cellular ROS, iron-based lipid peroxidation, and oxide accumulation [7–9]. Its relationship with various diseases has been established [10–12]. In experimental neurodegenerative disease, liver injury, renal failure, and intestinal disease models [13–16], ferroptosis inhibition has been shown to reduce clinical symptoms. For example, Qiu et al. reported that Nrf2 reduces ROS levels, thereby inhibiting ferroptosis and alleviating ALI induced by seawater drowning [17]. Li et al. reported that panaxydol inhibits ferroptosis during LPS-induced ALI via the KEAP1-NRF2/HO-1 pathway [18]. Dong et al. found that Nrf2 inhibits ferroptosis and protects against intestinal ischemia–reperfusion (IR)-induced acute lung injury [19]. The findings of these studies show that Nrf2 is an important negative regulator of ALI-related ferroptosis; ferroptosis promotes ALI progression, and its inhibition through Nrf2 activation provides a new therapeutic approach for ALI treatment. This prompted us to carry out research on Nrf2 activation and inhibitory drugs for ALI-related ferroptosis.

To date, studies have shown that natural flavonoids are essential for the prevention of diseases, especially those caused by inflammation and oxidative stress, such as ALI [20]. Obacunone (OB) is a natural limonoid that is widely found in citrus species and Rutaceae members and is notable because of its various biological activities, including anti-inflammatory and antioxidant properties [21–25]. Furthermore, recent studies have shown that OB attenuates liver fibrosis in mice by enhancing the antioxidant effects of GPX-4 [24]. However, the effects of OB against LPS-induced ALI have not yet been evaluated. In this study, we aimed to explore the protective effects of OB against LPS-induced ALI.

## Materials and methods

### Cell culture and reagents

Human bronchial epithelial cells (BEAS-2B cells, purchased from Procell, Wuhan, China) were cultured in RPMI-1640 (Hyclone, Hudson, NH, USA) and mixed medium supplemented with 10% fetal bovine serum, 200 U/mL penicillin G, and 200 mg/mL streptomycin in a 5% CO_2_ incubator at 37 °C for use in the subsequent experiments. Lipopolysaccharide (055:B5) was purchased from Solarbio (Beijing, China). Obacunone (AI3-37934, CCRIS 8657) was purchased from Selleck (Shanghai, China).

### Animals

Eight- to ten-week-old male wild-type (WT) mice (C57BL/6) were purchased from Beijing Weishanglide Biotechnology (Beijing, China), and were bred and raised in the animal room of Chengdu Medical College. All procedures involving animals were approved by the Animal Policy and Welfare Committee of Chengdu Medical College (CDYXY-2019036).

### Cell Counting Kit-8 assay

BEAS-2B cells were evenly distributed in the wells of a 96-well plate at a concentration of 3500 cells per well and incubated for further culture. Following the entry of cells into the logarithmic growth phase, the medium in the plate was discarded and 100 μL of culture medium containing different concentrations of OB was added. The same quantity of normal medium was added to cells in the control group, and three different wells were used for the different OB treatments. In some experiments, following treatment with LPS (10 μg/mL), the cells were treated with obacunone at different concentrations (0, 10, 20, and 40 μM). Thereafter, 10 μL of CCK8 solution was added to each well, and the plates were incubated at 37 °C for 1 h. Cell viability was measured using Cell Counting Kit-8 (Shanghai, China).

### Lactate dehydrogenase (LDH) assay

LDH level was measured according to the instructions of the LDH Cytotoxicity Assay Kit (Shanghai, China).

### Western blotting

For SDS-PAGE, a 12% separating gel, a 5% concentrated gel, an electrophoresis buffer, and a membrane transfer solution (precooled at 4 °C) were prepared and reserved until use. For SDS-PAGE electrophoresis, the samples were loaded according to the experimental requirements, the electrophoresis tank was filled with a mixed electrophoresis buffer, and the voltage was adjusted to 80 V and increased to 120 V after 30 min. A PVDF membrane was activated by immersion in methanol at a constant flow of 250 mA, and membrane transfer was carried out for 90 min. The PVDF membrane was removed and washed for 1 min. The membrane was blocked with 5% powdered skim milk at 37 °C for 2 h. The primary antibodies used in this experiment were mixed with a primary antibody diluent (1:1000) and incubated with the membrane overnight at 4 °C. Thereafter, the membrane was washed thrice with TBST and incubated at room temperature for 2 h with secondary antibodies diluted with TBST (1:4000). After incubation with the secondary antibodies, the membrane was developed and analyzed after being washed with TBST. The primary antibodies used for this assay were anti-Nrf2, anti-GPX4, anti-HO-1, anti-4-HNE, and anti-SLC7A11, which were purchased from Proteintech (Wuhan, China). The secondary antibodies used were also purchased from Proteintech.

### 5-Ethynyl-2′-deoxyuridine (EDU) assay

To evaluate cell proliferation, the cells were evenly distributed in the wells of 24-well plates at a density of 30,000 cells per well for culture. On day 2 of culture, the spent medium was discarded from the cell culture, replaced with EDU medium (10 μmol/L), and incubated for 4 h, according to the operating procedures of the EDU assay (Beyotime, Shanghai, China).

### Enzyme-linked immunosorbent assay (ELISA)

Cell culture supernatant and mouse bronchoalveolar lavage fluid (BALF) were collected, and the levels of chemokines and inflammation were determined using an ELISA kit (Shanghai, China) according to the manufacturer’s instructions. KL-6 (Shanghai, ml038403) and CRP (Shanghai, Mouse CRP ELISA Kit) were measured by ELISA according to the manufacturer’s instructions in plasma.

Lymphocytes (LYM) (%) and neutrophils (NEU) (%) were measured by XT-2000i (Japan, SYSMEX) in EDTA anticoagulation blood.

### Lung wet/dry (W/D) weight measurement

Lung samples were collected after LPS stimulation, immediately weighed (wet weight) after removal, and then dried in an oven at 80 °C for 48 h for dry weight measurement. Tissue edema was assessed by measuring the W/D ratio.

### Pathological examination of the lung tissues

The lung tissues were sampled, fixed with 4% paraformaldehyde at room temperature for 18–24 h, and then rinsed overnight with a fine stream of water. The lung tissues were dehydrated in an ethanol gradient, embedded in a wax block, and cut into 4–5-μm sections; after baking the sections at 60–62 °C for 3–4 h, they were dewaxed in xylene, immersed in an ethanol gradient, and washed with super-distilled water. For hematoxylin and eosin (HE) staining, after strictly following the HE staining instructions, the sections were sealed with neutral gum and observed under a light microscope. Protein expression in the lung tissues was evaluated by immunohistochemistry, and the main proteins evaluated were GPX4, 4-HNE, and Nrf2. The sections were heated in a microwave oven for 10 min for antigen repair. Histochemical pen circles were used to cover the tissue sections with 3% H_2_O_2_ at 37 °C for 20 min, and then the sections were washed with PBS. The sections were sealed with 10% goat serum at room temperature for 30 min, and then primary antibodies (1:50) were added and the sections were incubated overnight in a wet box at 4 °C. After washing the sections with PBS, universal secondary antibodies labeled with HRP were added and the samples were incubated at 37 °C for 2 h. After washing the tissue sections with PBS, fresh DAB was added for color rendering. Following this, the sections were restained with hematoxylin and the blue color was restored. The sections were dehydrated, sealed, and observed under a microscope for image collection.

### Malondialdehyde (MDA), superoxide dismutase (SOD), glutathione (GSH), and catalase (CAT) analyses in the lung tissues

The right lungs of the mice were resected after intraperitoneal LPS injection. The lung tissue homogenates were dissolved in the extraction buffer. MDA, SOD, GSH, and CAT levels were measured according to the manufacturer’s instructions of the corresponding kits (Beyotime, Shanghai, China).

### Detection of Fe^2+^

Cell samples were washed twice in cold PBS; then 200 μL lysate was added per well, and the plate was placed on a shaker for 2 h. Then, mix A and the standard were diluted according to the manufacturer’s instructions. Samples were mixed in different groups and incubated at 60 °C for 1 h. The samples were cooled to 28 °C, and the droplets on the top of the tube wall were centrifuged to the bottom. After 30 μL of iron ion detection agent was added and the sample was incubated at room temperature for 30 min, 200 μL of the solution was added to a 96-well plate and the absorbance was measured at 550 nm. Finally, the standard curve was plotted and the iron ion concentration was calculated.

### Immunofluorescence assay

The prepared cell slides were washed thrice with PBS and fixed with 4% paraformaldehyde at room temperature for 30 min, then with 0.3% Triton X‐100 for 3 min. After washing the slides with PBS, the cells were sealed with 10% BSA for 30 min and incubated with primary antibodies (1:300) at 4 °C overnight. The following day, after repeated washing, the cells were incubated with fluorescent secondary antibodies (1:300) at room temperature for 60 min in the dark. DAPI (1:4000) staining was performed for 8 min before fluorescence microscopy.

### Terminal deoxynucleotidyl transferase dUTP nick end labeling (TUNEL) lung tissue section staining

Tissue sections of 4–5 μm were soaked in xylene at room temperature, dehydrated in an ethanol gradient, washed with PBS, and circled with histochemical strokes to facilitate the subsequent operations. Next, 100 μL of Proteinase K working solution (PBS 1:9 dilution) was added to all samples, after which the samples were incubated at 37 °C for 20 min. After washing the sections with PBS, 0.3% Triton X-100 was added and the samples were incubated at room temperature for 20 min. After washing the samples again with PBS, 50 μL of Slides Bration buffer was added, and the sections were subsequently incubated with equilibration buffer at 37 °C for 10 min. As much Bration buffer as possible was removed from the sections, and recombinant TdT enzyme, FITC-12-DUTP Labeling Mix, and Bration buffer were added on the sections at a ratio of 1:5:50 µL. The TdT incubation buffer was proportionally configured and added onto the sections until they were covered, after which the sections were incubated at 37 °C for 1 h, then stained with DAPI (1:4000) at room temperature for 8 min. The samples were analyzed under a fluorescence microscope immediately after sealing. Of note, the sample was not dried throughout the experiment to avoid the effect of light. The TUNEL kit was purchased from Servicebio (Wuhan, China).

### Fluorescence quantitative PCR

The cells were lysed in each dish using TRIzol reagent (TSP413; TSINGKE, Shanghai, China), and the total RNA was extracted according to the manufacturer’s instructions. The RT6 cDNA synthesis kit (TSK302M, TSINGKE) was used to reverse transcribe the RNA into cDNA (stored at −80 °C). Real-time quantitative PCR was performed using the CFX96 real-time system and SYBR Green I (TSE202, TSINGKE). The primer sequences were as follows: NQO1: forward GGGTGCCAGCCATTCTGAAAGG, reverse CCCAGTGGTGATAGAAAGCAAGGTC; HO-1: forward CACAGATGGCGTCACTTCGTCAG, reverse GAGGAGCGGTGTCTGGGATGAG; GPX4: forward CCCGATACGCTGAGTGTGGTTTG, reverse TCTTCGTTACTCCCTGGCTCCTG; Nrf2: forward TAAAGCACAGCCAGCACATTCTCC, reverse TGATGACCAGGACTCACGGGAAC; and GAPDH: forward ACAGTCAGCCGCATCTTC, reverse CTCCGACCTTCACCTTCC.

### Flow cytometry

For the cell death assay, BEAS-2B cells were collected and initially stained with 7-aminoactinomycin D (7-AAD; 2 μg/mL in PBS; KeyGEN, Jiangsu, China) for 20 min, and washed thrice. A portion of the cells was then analyzed using a flow cytometer (FACSCalibur, Becton–Dickinson, Franklin Lakes, NJ, USA), and the data were collected for analysis.

### Statistical analysis

All experiments were independently repeated at least three times, and all animals were randomly assigned to the experimental groups. A one-way ANOVA or paired *t-*test was used to determine statistical significance between the groups. Statistical significance was set at *P* < 0.05. Statistical analyses were performed using GraphPad Prism 7.0 (GraphPad, San Diego, CA, USA).

## Results

### Treatment with OB alleviated LPS-induced BEAS-2B cell injury

**Fig. 1:**
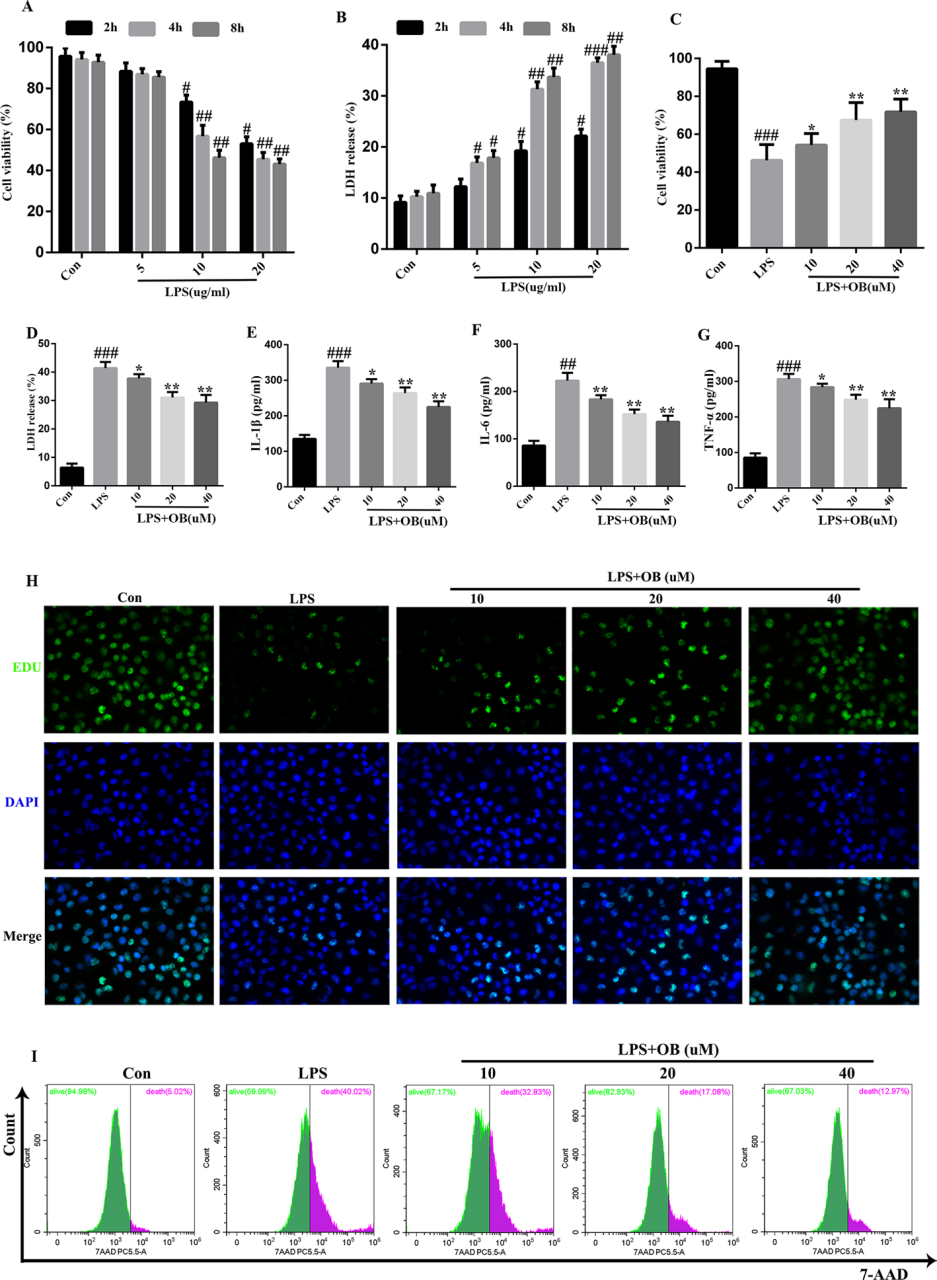
Protective effects of obacunone against lipopolysaccharide (LPS)-induced BEAS-2B damage. **A**, **B** LPS treatment decreased cell viability and increased lactate dehydrogenase (LDH) release as determined using Cell Counting Kit-8 (CCK-8) **(A)** and LDH **(B)** assays. **C–G** Obacunone (OB) increased cell viability **(C)**, and inhibited LDH **(D)**, IL-1β **(E)**, IL-6 **(F)**, and TNF-α **(G)** release in a dose-dependent manner (0, 10, 20, or 40 μM) following LPS treatment (10 μg/mL). **H** Representative EDU assay fluorescence images. **I** Representative flow cytometric images. Similar results were obtained from three independent experiments. All data are presented as mean ± standard error of the mean (SEM) (*n* = 6 for each group). ^#^/*P < 0.05, ^##^/**P < 0.01, ^###^/***P < 0.001, ^#^ versus control group; * versus LPS group

First, we established an LPS-induced BEAS-2B cell injury model. As shown in Fig. 1, treatment with LPS reduced cell viability and increased LDH release in BEAS-2B cells in a dose-dependent manner at 2, 4, and 8 h (Fig. 1A, B). From these preliminary findings, we chose an LPS dose of 10 μg/mL and a treatment duration of 4 h for all subsequent experiments. To evaluate the protective effects of OB against ALI, BEAS-2B cells were pretreated with different concentrations of OB for 4 h [26] before LPS treatment. We found that cell viability increased with the increase in OB concentration (Fig. 1C) and that OB inhibited LDH release in a dose-dependent manner (Fig. 1D). In addition, the secretion of the inflammatory cytokines, IL-1β, IL-6, and TNF-α, was decreased in a dose-dependent manner (Fig. 1E–G). EDU staining and flow cytometry showed that treatment with OB effectively promoted cell survival (Fig. 1H–I). Therefore, we chose an OB dose of 20 μM and a treatment duration of 4 h for all subsequent experiments. Collectively, these results suggest that OB has protective effects against LPS-induced BEAS-2B cell injury.

### Treatment with OB alleviated LPS-induced ALI in mice

**Fig. 2:**
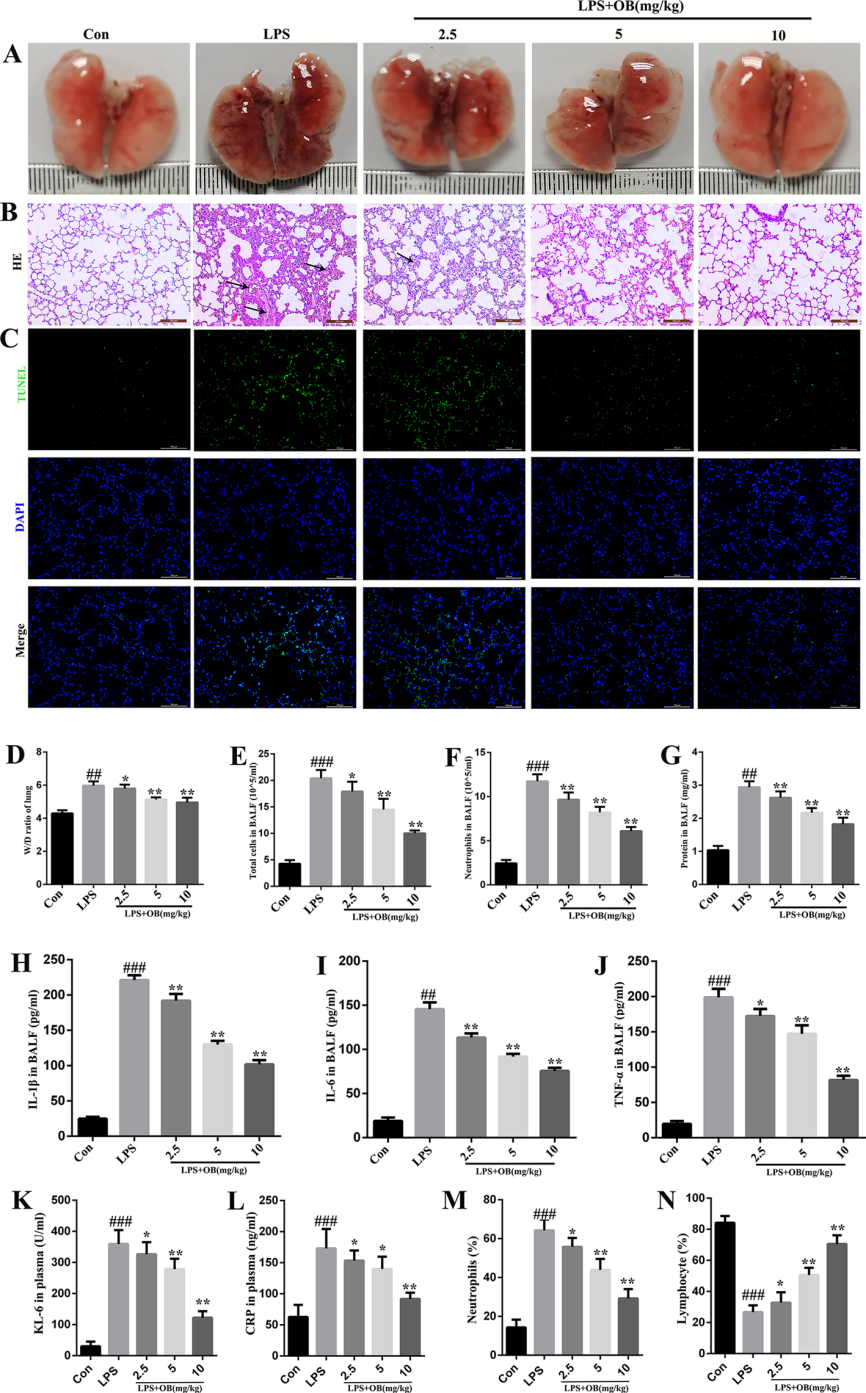
Protective effects of obacunone (OB) against lipopolysaccharide (LPS)-induced acute lung injury (ALI) in mice. **A** Representative images of mouse lung tissues. Lung tissues (*n* = 6) from each experimental group were processed for histological evaluation following the LPS challenge. **B** Representative hematoxylin and eosin (HE) staining images of the lung tissues. **C** Representative terminal deoxynucleotidyl transferase dUTP nick end labeling (TUNEL) fluorescence images of the lung tissues. **D** Lung wet weight/dry (W/D) weight ratio. **E–G** Total cell and neutrophil count and protein concentration in bronchoalveolar lavage fluid (BALF). **H–J** IL-1β, IL-6, and TNF-α concentrations in the BALF. **K, L** KL-6 and CRP in plasma. **M, N** LYM (%) and NEU (%) in EDTA anticoagulation blood. Similar results were obtained from three independent experiments. All data are presented as mean ± SEM (*n* = 6 for each group). ^#^/*P < 0.05, ^##^/**P < 0.01, ^###^/***P < 0.001, ^#^ versus control group; * versus LPS group

To further evaluate the effects of OB against LPS-induced ALI in mice, the mice were intraperitoneally injected with OB (2.5, 5, and 10 mg/kg) for 48 h, and then intraperitoneally injected with LPS (10 mg/kg) for 7 h to induce ALI (based on previous studies) [22, 27]. As shown in Fig. 2A, the lungs of LPS-induced mice showed significant edema and hyperemia compared with control mice. In addition, OB reduced the LPS-induced loss of ALI lung tissue structure loss (Fig. 2B), apoptosis injury (Fig. 2C), and edema (Fig. 2D); reduced the total number of cells and neutrophils, protein content, and IL-6, lL-1 β, and TNF-α levels in mouse BALF (Fig. 2E–J); reduced KL-6 (Fig. 2K), CRP (Fig. 2L) and neutrophils (%) (Fig. 2M); and increased lymphocytes (%) (Fig. 2N). Collectively, our results indicated that OB significantly improved the inflammatory response and pathological changes in the lung tissues of LPS-induced mice. Therefore, ALI was induced in the model group only by LPS (10 mg/kg) intraperitoneal injection for 7 h. The mice in the experimental group were intraperitoneally administered OB (10 mg/kg) for 48 h, followed by intraperitoneal LPS (10 mg/kg) injection for 7 h, to induce ALI for the subsequent experiments.

### Treatment with OB reduced oxidative stress during LPS-induced ALI

**Fig. 3:**
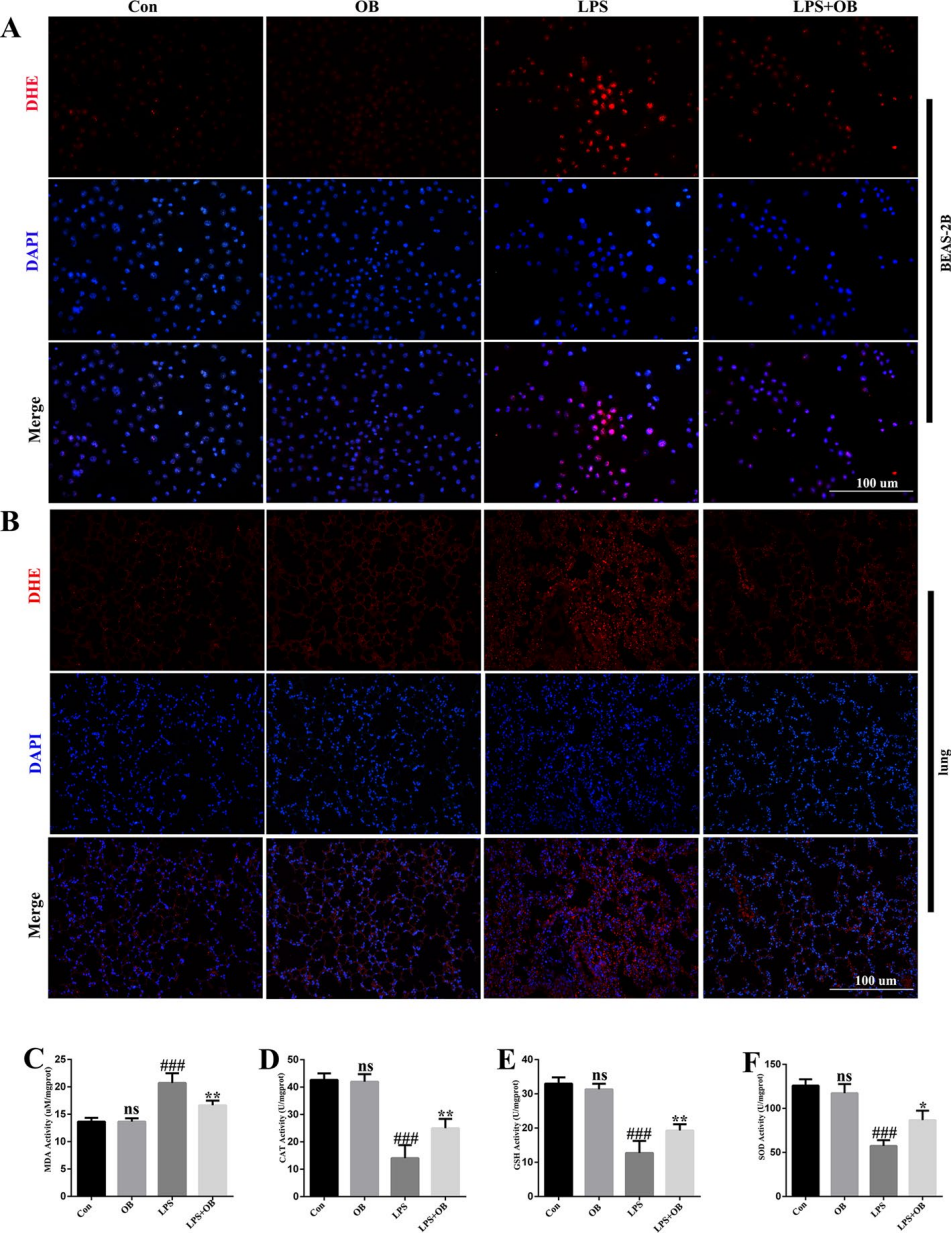
Effects of obacunone against lipopolysaccharide (LPS)-triggered oxidative stress in induced acute lung injury (ALI). **A** Representative dihydroethidium (DHE) fluorescence images of BEAS-2B cells. **B** Representative DHE fluorescence images of the mouse lung tissues. **C–F** Activities of malondialdehyde (MDA), catalase (CAT), glutathione (GSH), and superoxide dismutase (SOD) in the mouse lung tissues. Similar results were obtained from three independent experiments. All data are presented as mean ± SEM (*n* = 6 for each group). ^#^/*P < 0.05, ^##^/**P < 0.01, ^###^/***P < 0.001, ^#^ versus control group; * versus LPS group; ns, no significant difference between control and OB group

Oxidative damage significantly contributes to the development of LPS-induced ALI, and we investigated whether pretreatment with OB can inhibit LPS-induced oxidative stress. We found that pretreatment with OB significantly reduced LPS-induced ROS generation in BEAS-2B cells and mice (Fig. 3A and B). The MDA level in mice treated with OB was significantly lower than that in mice in the LPS group (Fig. 3C). In addition, OB restored the activities of CAT, GSH, and SOD, which were inhibited by LPS stimulation (Fig. 3D–F). These results showed that OB could protect against LPS-induced ALI by decreasing the ROS and MDA levels and by increasing the CAT, GSH, and SOD levels.

### OB inhibited ferroptosis and alleviated LPS-induced BEAS-2B injury and ALI

**Fig. 4:**
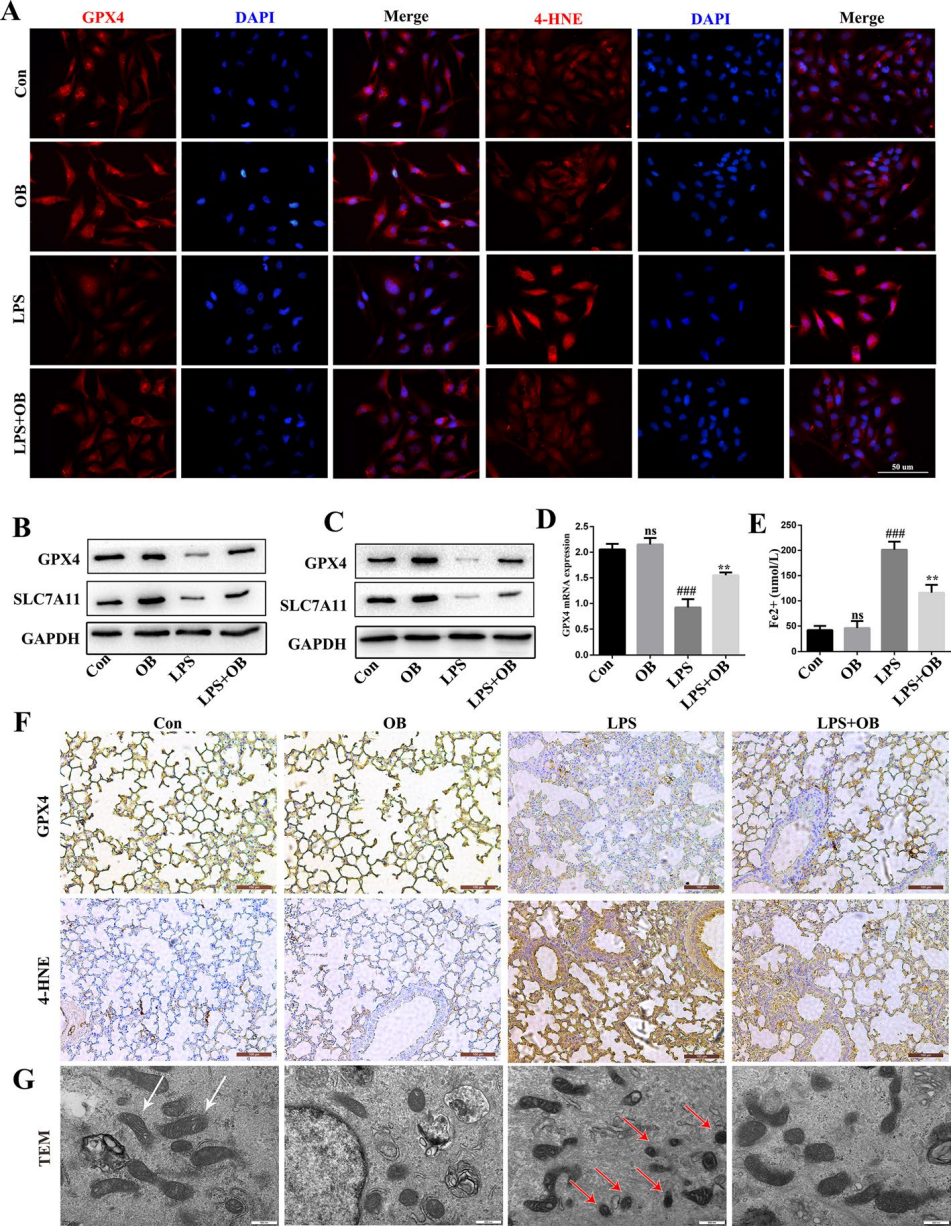
Ferroptosis was upregulated during lipopolysaccharide (LPS)-induced injury in vivo and in vitro. **A** Representative immunofluorescence images of GPX4 and 4-HNE in BEAS-2B cells. **B, C** Western blotting of GPX4 and SLC7A11 in mice **(B)** and BEAS-2B cells **(C)**. **D** Expression levels of *GPX4* in BEAS-2B cells as determined by RT-qPCR. **E** Expression level of Fe^2+^ in BEAS-2B cells. **F** Immunohistochemistry-based images of GPX4 and 4-HNE in the lung tissues. **G** Transmission electron microscopy (TEM) of ferroptosis in BEAS-2B cells. Similar results were obtained from three independent experiments. All data are presented as mean ± SEM (*n* = 6 for each group). ^##^/**P < 0.01, ^###^/***P < 0.001, ^#^ versus control group; * versus LPS group; ns, no significant difference between control and OB group

Ferroptosis is caused by iron accumulation and lipid peroxidation, and is characterized by mitochondrial contraction. In the LPS-induced ALI model, the ferroptosis level in the lung tissues was assessed by measuring Fe^2+^, GPX4, 4-HNE, and SLC7A11 levels. As shown in Fig. 4, GPX4 and its mRNA levels were significantly decreased and 4-HNE and Fe^2+^ levels were significantly increased in BEAS-2B cells in the LPS group, compared with those in the control group (Fig. 4A, D, and E). In addition, GPX4 and SLC7A11 levels were increased in both BEAS-2B cells (Fig. 4B) and mouse lung tissues (Fig. 4C) following OB treatment. Immunohistochemical staining and transmission electron microscopy (TEM) analysis showed that the GPX4 level significantly decreased, and the 4-HNE level significantly increased (Fig. 4F) in mice in the LPS group. They also showed that there was significant mitochondrial contraction (Fig. 4G) in mice in this group. These findings suggest that OB may ameliorate LPS-induced ALI by inhibiting ferroptosis.

### Ferroptosis inhibitor, Fer-1, alleviated LPS-induced ALI in mice

**Fig. 5:**
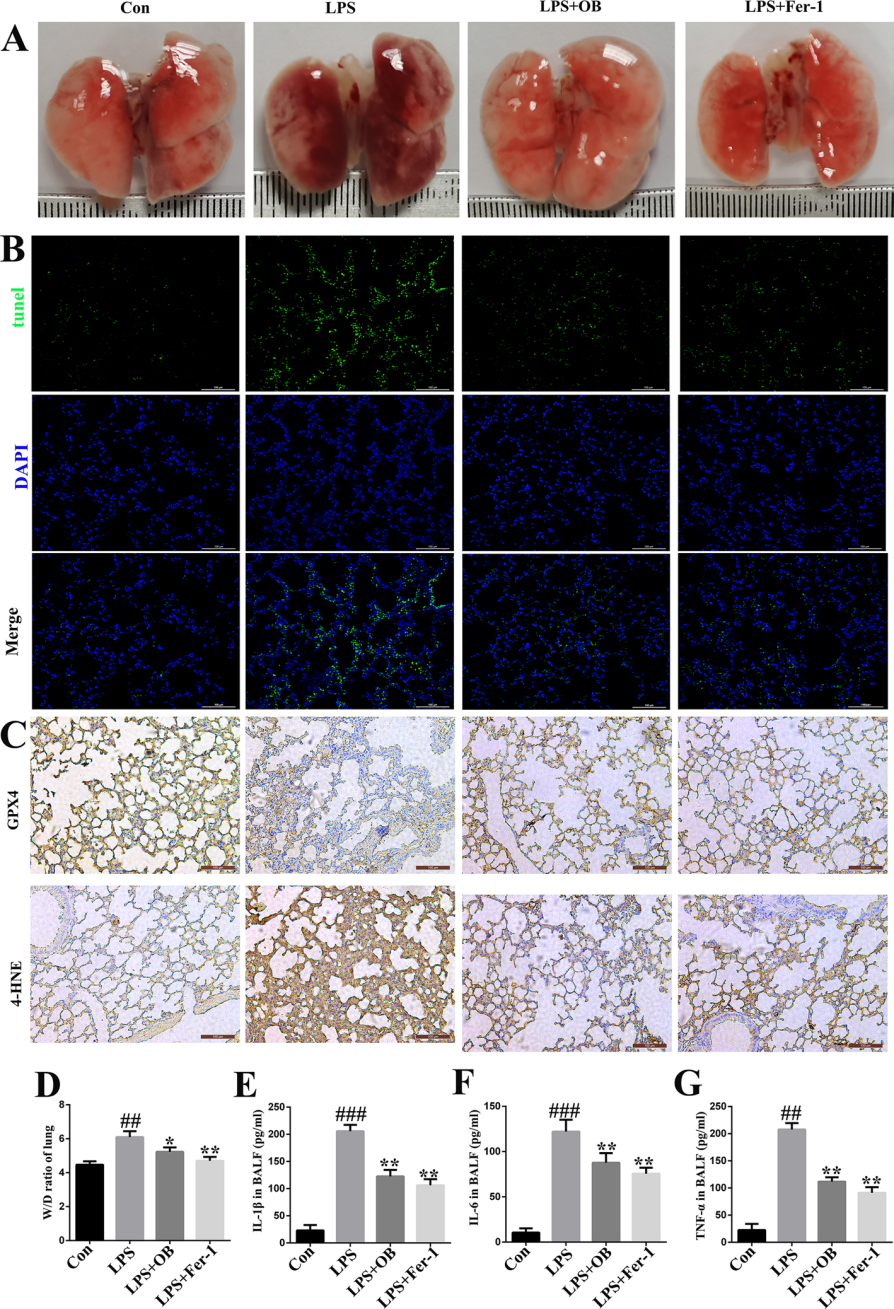
Effects of the ferroptosis inhibitor on lipopolysaccharide (LPS)-induced acute lung injury (ALI). **A** Representative images of the mouse lung tissues. **B** Representative terminal deoxynucleotidyl transferase dUTP nick end labeling (TUNEL) fluorescence images of the lung tissues. **C** Representative immunohistochemistry-based images of GPX4 and 4-HNE in the lung tissues. **D** Lung wet weight/dry (W/D) weight ratio. **E–G** Concentrations of IL-1β, IL-6, and TNF-α in bronchoalveolar lavage fluid (BALF). Similar results were obtained from three independent experiments. All data are presented as mean ± SEM (*n* = 6 for each group). ^#^/*P < 0.05, ^##^/**P < 0.01, ^###^/***P < 0.001, ^#^ versus control group; * versus LPS group

To further evaluate the role of ferroptosis in LPS-induced ALI, the mice were pretreated with a ferroptosis inhibitor (Fer-1: 5 mg/kg) for 24 h and the association between ferroptosis and inflammation was assessed (based on a previous study) [28]. We found that OB significantly inhibited ferroptosis and inflammation in the LPS-induced ALI mouse model, and this was similar to the effect achieved with Fe-1 treatment. These findings suggest that ferroptosis mediates inflammation during LPS-induced ALI, and that OB ameliorates LPS-induced inflammation by inhibiting ferroptosis (Fig. 5).

### OB upregulated Nrf2 expression in LPS-treated BEAS-2B cells and in the ALI mouse model

**Fig. 6:**
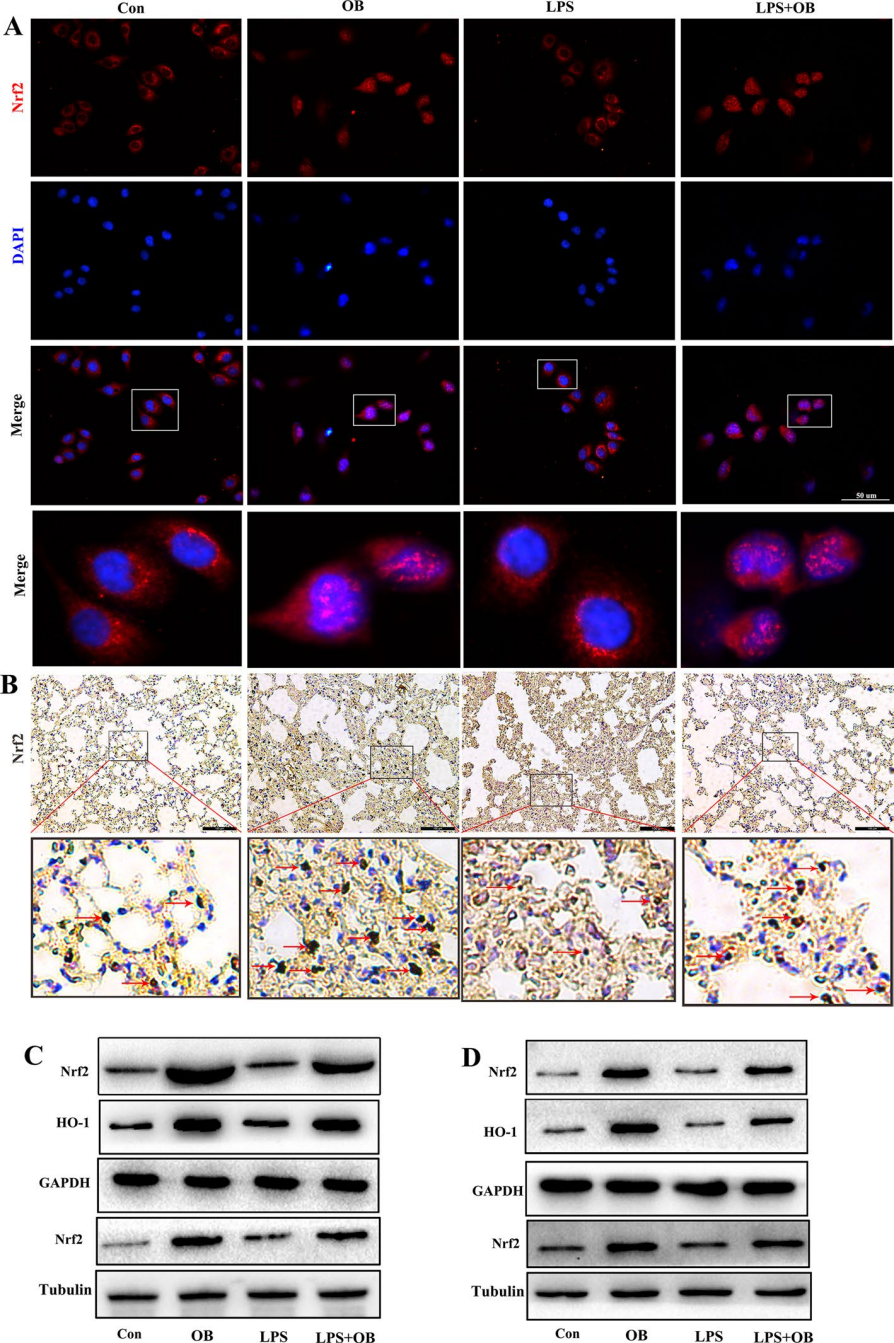
Obacunone (OB) upregulated Nrf2 level in lipopolysaccharide (LPS)-induced acute lung injury (ALI) models. **A** Representative immunofluorescence images of Nrf2 in BEAS-2B cells. **B** Representative immunohistochemistry-based images of Nrf2 in the mouse lung tissues. **C, D** Nrf2 and HO-1 level in BEAS-2B cells **(C)** and the mouse lung tissues **(D)** as determined by western blotting. Similar results were obtained from three independent experiments. All data are presented as mean ± SEM (*n* = 6 for each group). ^##^/**P < 0.01, ^###^/***P < 0.001, # versus control group; * versus LPS group

To explore the mechanisms underlying the protective effects of OB against LPS-induced BEAS-2B cell injury and ALI, the Nrf2 level and locations were analyzed through cell immunofluorescence (Fig. 6A), immunohistochemical analysis of the lung tissues (Fig. 6B), and western blotting (Fig. 6C, D). Treatment with OB promoted Nrf2 entry into the nucleus and upregulated Nrf2 expression, thus indicating that the protective effects of OB against LPS-induced ALI may be related to Nrf2 upregulation.

### OB inhibited ferroptosis through Nrf2 activation

**Fig. 7:**
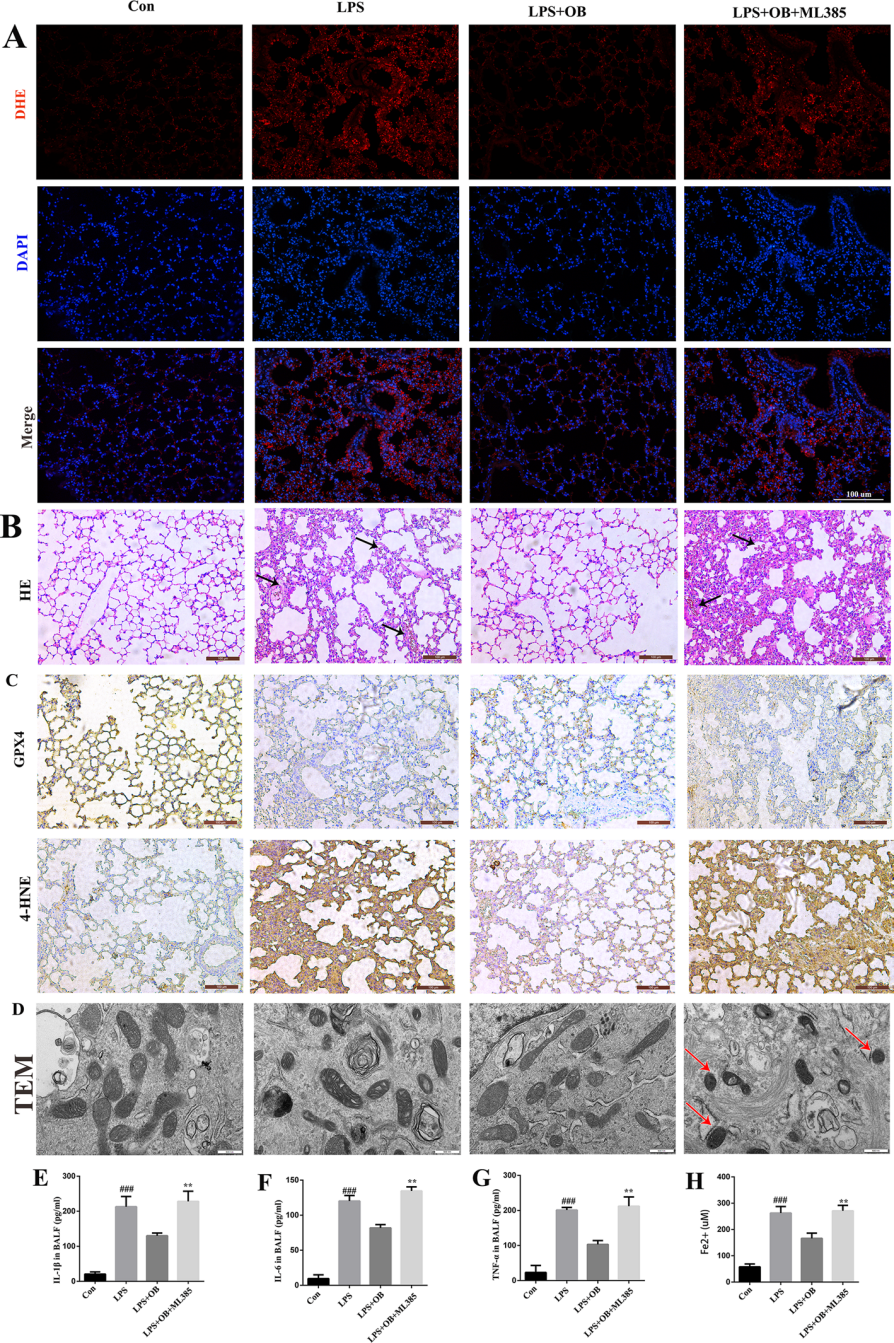
Obacunone (OB) inhibited ferroptosis by activating nuclear factor-2 associated factor 2 (Nrf2). **A** Representative dihydroethidium (DHE) fluorescence images of the mouse lung tissues. **B** Representative hematoxylin and eosin (HE) staining images of the lung tissues. **C** Representative immunohistochemistry-based images of GPX4 and 4-HNE in the lung tissues. **D** Representative transmission electron microscopy (TEM) images of ferroptosis in BEAS-2B cells. **E–G** IL-1β, IL-6, and TNF-α concentrations in bronchoalveolar lavage fluid (BALF). **H** Fe^2+^ level in BEAS-2B cells. Similar results were obtained from three independent experiments. All data are presented as mean ± SEM (*n* = 6 for each group). ^##^/**P < 0.01, ^###^/***P < 0.001, ^#^ versus control group; * versus LPS + OB group

To further verify whether OB elicits its protective effects against ALI through Nrf2 activation, mice were pretreated with an Nrf2 inhibitor (ML385 30 mg/kg) for 2 h (based on a previous study) [29], and subsequently stimulated with OB. As shown in Fig. 7, ML385 significantly reversed the protective effects of OB against ferroptosis, oxidative stress, and the inflammatory response. These findings suggest that Nrf2 plays an important role in the development of LPS-induced ALI, and that OB may ameliorate LPS-induced ALI by activating Nrf2, thereby reducing oxidative stress and inhibiting ferroptosis.

### OB reduced Nrf2 ubiquitinated proteasome degradation

**Fig. 8:**
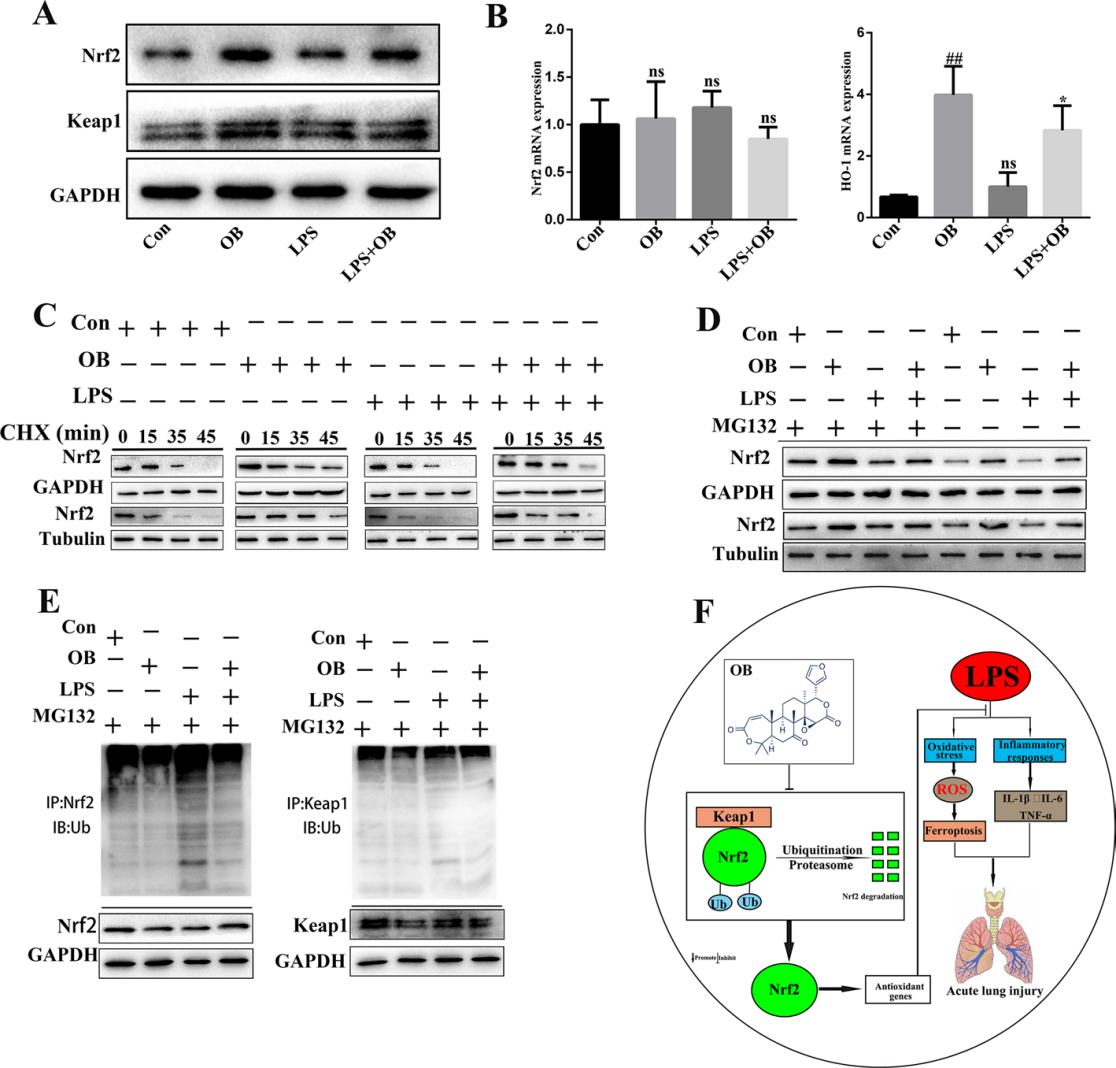
Obacunone (OB) reduced nuclear factor-2 associated factor 2 (Nrf2) ubiquitinated proteasome degradation. **A** Expression of Nrf2 and Keap1 in BEAS-2B cells as determined by western blotting. **B** Expression levels of *Nrf2* and* HO-1* in BEAS-2B cells as determined by RT-qPCR. **C** BEAS-2B cells were treated with 50 μg/mL cycloheximide (CHX) and incubated for a specified period. Nrf2 level in BEAS-2B cells as determined by western blotting. **D** BEAS-2B cells were either left untreated or treated with 10 μM MG132 for 4 h to inhibit the degradation of ubiquitinated proteins. Nrf2 level was determined by western blotting. **E** BEAS-2B cells were treated with 10 μM MG132 for 4 h, and endogenous ubiquitin-bound Nrf2 and Keap1 were detected. Cell lysates were immunoprecipitated using anti-NRF2 and anti-Keap1 antibodies and imprinted with anti-ubiquitin antibodies. **F** Diagram showing the mechanism of action of OB. Similar results were obtained from three independent experiments. All data are presented as mean ± SEM (*n* = 6 for each group). ^#^/*P < 0.05, ^##^/**P < 0.01, ^#^ versus control group; * versus LPS group; ns, no significant difference between control and LPS group

The Keap1–Nrf2 signaling pathway is the main antioxidant defense mechanism against environmental stress-induced damage. We evaluated the protein levels of Keap1 and Nrf2 and the mRNA levels of *Nrf2* and *HO-1* in the presence or absence of OB, and found that there was no significant difference in the Keap1 protein levels and *Nrf2* mRNA levels irrespective of OB treatment (Fig. 8A and B). This prompted us to speculate that OB might affect Nrf2 protein level. Therefore, we treated BEAS-2B cells with the Nrf2 protein synthesis inhibitor, cycloheximide (CHX: 50 μg/mL), and found that it accelerated LPS-induced Nfr2 degradation, whereas OB reduced Nrf2 degradation (Fig. 8C). To determine whether OB inhibits Nrf2 ubiquitination and reduces its degradation through the ubiquitin–proteasome pathway, LPS-induced BEAS-2B cells were treated with the proteasome inhibitor, MG132 (based on a previous study) [22]. We found that MG132 restored Nrf2 protein levels in LPS-treated BEAS-2B cells (Fig. 8D). In addition, the ubiquitin analysis showed increased rates of ubiquitin and Nrf2 binding in LPS-induced BEAS-2B cells, whereas OB pretreatment reduced the rate of ubiquitin and Nrf2 binding (Fig. 8E). Collectively, these findings suggest that OB stabilizes Nrf2 by inhibiting its degradation by ubiquitin–proteasome.

## Discussion

ALI and ARDS can cause severe lung diseases or induce the pulmonary manifestations of multiple organ dysfunction syndrome; they can also induce uncontrolled and self-amplified lung inflammation [30, 31]. As these diseases are associated with the high morbidity and mortality rates in critically ill patients, they constitute a major public health burden; moreover, they have no effective patient-specific treatments [32, 33]. While significant advances have been made in understanding the pathogenesis of these diseases, clinically available treatments are still limited and patients have a poor quality of life. Therefore, developing new drugs or treatment strategies for ALI or ARDS is crucial. Here, we demonstrated that OB effectively enhances Nrf2 activity and blocks ferroptosis, thereby identifying it as a potential therapeutic drug for ALI.

OB has attracted attention owing to its various biological activities, such as its anti-inflammatory and antioxidant properties [21–25]. However, its potential for use in the treatment of ALI has not been clarified. To date, studies have shown that ferroptosis promotes ALI progression [34–37]. In this study, we investigated the role of ferroptosis in LPS-induced ALI. Our results suggest that LPS can induce ferroptosis in the lung tissues and cells both in vitro and in vivo and that ferroptosis inhibitors exhibit therapeutic effects against LPS-induced ALI, thereby providing new insights into the cell death pathways associated with LPS-induced ALI. For example, Wang et al. [38] showed that liver ferroptosis is an important inflammatory trigger for steatohepatitis, whereas Zhou et al. [39] found that intestinal SIRT1 defense alleviates defects in ethanol-induced hepatitis mouse models by alleviating ferroptosis. Our findings imply that OB reduced lung W/D ratio, protein levels, and pro-inflammatory factor release, and the levels of these effects almost correlated with the severity of ALI. In addition, OB decreased LPS-induced excessive ROS accumulation and MDA formation in the lungs, while increasing the production of the antioxidant enzymes SOD and GSH. Interestingly, we found that OB can effectively inhibit LPS-induced pulmonary pathology, pneumonia, and edema in vivo. Ferroptosis was found to occur during LPS-induced ALI, and this was consistent with the findings of Liu et al. [34]; additionally, OB significantly reduced LPS-induced ferroptosis. These findings indicate that OB inhibited LPS-induced ferroptosis and ROS production in vivo and in vitro and may have ameliorated LPS-induced ALI by inhibiting ferroptosis.

We further investigated the potential mechanisms underlying the effects of OB against LPS-induced ALI. Some studies have shown that Nrf2 is a signal coordinator, attenuating LPS-induced ALI by inhibiting inflammation and oxidative stress [40]. Under normal conditions, Nrf2 binds to Kelch-like ECH-associated protein 1 (Keap1), which is then ubiquitinated by a Cul3-based E3 ligase and degraded. In response to oxidative stress, it is activated, dissociated from Keap1, and translocated to the nucleus, thereby promoting SOD, CAT, and GSH expression to increase cellular resistance to oxidative stress [41–44]. As intracellular protein levels are determined by the balance between protein synthesis and degradation, we evaluated the parameters related to Nrf2 in BEAS-2B cells following OB treatment. The qPCR analysis showed that OB significantly increased the HO-1 mRNA levels, which are regulated by Nrf2; however, no significant change in the Nrf2 mRNA level was observed, prompting us to speculate that OB might affect Nrf2 degradation. Therefore, BEAS-2B cells were treated with CHX, a protein synthesis inhibitor, and the Nrf2 degradation rate was analyzed. We found that treatment with OB reduced Nrf2 degradation, thereby extending its half-life. To determine whether OB reduced Nrf2 degradation by reducing its ubiquitination through the ubiquitin–proteasome pathway, we treated BEAS-2B cells with the proteasome inhibitor MG132 and found that it restored Nrf2 protein levels in OB-treated EBAS-2B cells. Therefore, targeted Nrf2 activation can prevent the occurrence and development of ALI.

A significant curative effect and few side effects are the main references for clinical drug selection. Generally, as the drug concentration increases the treatment effect, the side effects will also increase accordingly; this leads to the limited value of many drugs. It is worth noting that our study showed that a certain dose of OB exerted no significant toxicity, both in vivo and in vitro. Although many candidate molecular targets and agents have been proposed to treat ALI (for example, lipid-core nanocapsules and Ge-Gen-Qin-Lian were found to ameliorate LPS-induced ALI [45, 46]), the development of therapeutic strategies to improve ALI treatment outcomes remains limited. Previous studies and clinical trials have shown that oxidative stress is closely associated with the development and progression of ALI; Nrf2 plays a key role in the regulation of oxidative stress. OB, as a novel agonist of Nrf2, can resist the oxidative stress caused by LPS-induced ALI and is expected to be a prospective therapeutic option to alleviate ALI in clinical practice.

## Conclusions

In summary, our study showed that OB exhibits significant protective effects against LPS-induced ALI in mice, and that it significantly decreases LPS-induced histopathological changes, inflammatory cell count, and the W/D weight ratio. OB can improve antioxidant capacity in the lung tissues, reduce LPS-induced ferroptosis, and stabilize Nrf2 by reducing its degradation by ubiquitinated proteasomes, thereby eliciting protective effects against LPS-induced ALI. Our findings suggest that OB has a potential for use in the prevention of ALI and that it has broad application prospects.

## Data Availability

The datasets used and/or analyzed during the current study are available from the corresponding author on reasonable request.

## Abbreviations

ALI: Acute lung injury
OB: Obacunone
Nrf2: Nuclear factor-2 associated factor 2
LPS: Lipopolysaccharide
MDA: Malondialdehyde
ARDS: Acute respiratory dysfunction syndrome
ROS: Reactive oxygen species
W/D: Lung wet/dry weight ratio
SOD: Superoxide dismutase
GSH: Glutathione
CAT: Catalase
CHX: Cycloheximide
